# Promoting Health Equity: A New Challenge for Frontiers in Public Health

**DOI:** 10.3389/fpubh.2013.00027

**Published:** 2013-08-23

**Authors:** Will R. Ross

**Affiliations:** ^1^Department of Medicine, Renal Division, Washington University School of Medicine, Saint Louis, MO, USA

**Keywords:** health equity, medical education, public health workforce, health literacy, diversity

Public Health Education and Promotion, a new section of the journal Frontiers in Public Health, provides a refreshing forum for those readers who have traversed the familiar landscape of near-intractable health inequities on a national and global level and desire new knowledge on how best to ameliorate health inequities and the social factors that perpetuate them. The new “frontier” in public health hopefully heralds an era of increased and appropriate resource allocation for interventions that improve public health education and consequently begins to shift the needle toward health equity. This article highlights a series of approaches that, asserting an ecological model, can promote and sustain health equity by:

- Providing an easily accessible repository of public health data and health information that promotes health literacy at a population level.
- Elevating public health research on health equity, aligning it with the best clinical and clinical translational research at leading institutions.
- Identifying strategies to ameliorate health inequity, primarily by promoting evidence-based, public health pedagogy in all institutions of higher education while advocating for the development of a diverse public health workforce.

## Health Literacy and Disparities

Poor health literacy has been strongly linked to health disparities (1–4). Health literacy is defined in the Institute of Medicine (IOM) report Health Literacy: A Prescription to End Confusion as “the degree to which individuals have the capacity to obtain, process, and understand basic health information and services needed to make appropriate health decisions” (5). Health literacy goes beyond a narrow concept of health education and individual behavior-oriented communication and addresses the environmental, political, and social determinants of health. The body of health literacy research and practice has expanded remarkably in the past decade; however we are still in the nascent stages of understanding the interface between health literacy and public health. Kristen Sorenson and colleagues offered a conceptual framework that integrates the “medical” conceptualization of health literacy with the broader “public health” perspective (6). Their model illustrates the necessary interface between disease promotion and social determinants to address public health literacy, with the individual health promotion found in traditional health literacy. It is now incumbent upon leading scientific journals to provide concrete examples of how health literacy approaches can be used to improve community and population health and reduce health disparities. Readers will have the opportunity to review the current field of public health literacy and health disparities in more detail in a forthcoming edition of this journal.

## Funding Public Health Research

Public health education research needs to be funded at a level commensurate with the urgency to reduce the burden of chronic diseases and health inequity worldwide, and Public Health Education and Promotion has to be at the forefront of disseminating that research to a broad audience. Public health education research has to specifically address the socioeconomic, environmental, and behavioral determinants of disease (7), which has to occur in a collaborative manner among the leading academic medical centers, schools of public health, and institutions such as the National Institutes of Health (NIH) and Centers for Disease Control and Prevention (CDC). The Affordable Care Act (8) recently ushered in a new level of research collaboration through the establishment of the Prevention and Public Health Fund, which has invested substantially in evidence-based activities including research.

According to the Department of Health and Human Services, the CDC has continued support for its Prevention Research Centers program (9). This effort directs a national network of 37 academic research centers, at either a school of public health or a medical school with a preventive medicine residency program. The centers are committed to conducting prevention research and are leaders in translating research results into public health practice. These centers have rich capacity for the community-based, participatory prevention research needed to understand the major community changes that can prevent and control chronic diseases. The Agency for Healthcare Research and Quality (AHRQ) has also established Centers for Excellence in Clinical Preventive Services in Illinois, North Carolina, and Colorado which support the HHS National Prevention Strategy by developing evidence around the most efficient and effective ways primary care health systems can deliver clinical preventive services (10). These and other examples of community-based participatory prevention research should be extensively highlighted to promote more widespread adoption.

## Integrating Public Health in Medical Education

As outlined by the CDC and the NIH Institute for Minority Health, the future health of the nation will be determined to a large extent by how effectively we work with communities to eliminate health disparities among those populations experiencing a disproportionate burden of disease, disability, and death (11). Nonetheless, health professional schools have produced a practitioner workforce more educated in specific disciplines and lacking in formal public health training. In September 2010 the Association of American Medical Colleges (AAMC) and the CDC sponsored a workshop (12), “Patients and Populations: Public Health in Medical Education” which provided a framework for integrating public health content into medical school curricula. It is timely that schools of medicine and allied health provide members of the future physician workforce the knowledge, skills, and attitudes to address health disparities in partnership with community health and public health workers.

Unquestionably, a reduction in health disparities will require more interdisciplinary approaches, with a more active interface between public health programs and health professional schools, coupled with a systematic approach that aligns governmental and non-governmental agencies to specifically address health equity. Indeed, the resolution of this country's persistent health inequities is intrinsically linked to enhanced instruction in public health in our schools of medicine, public health, and allied health (13, 14). Such comprehensive health disparities curricula have been infrequent in contemporary health professional schools and organizations (15). The Liaison Committee for Graduate Medical Education (LCGME) adopted standards for cross-cultural education as early as 2002 (16), and both the AAMC and IOM (17, 18) have provided recommendations for health disparities education. However, health disparities curriculum in health professional schools may fail to evaluate knowledge, skills, and attitudes over time and are less likely to be linked to improved patient heath status and greater community benefit.

While there is continued interest in courses on healthcare delivery and healthcare systems, there may be less in other appropriate areas like occupational health and medicine. Health policy development is required in <70% of medical schools despite the fact that 77% of polled Americans most trust physicians to reform the health system (19, 20). These data follow the 1998 Healthy People Curriculum Task Force (21) which recommended that population health perspectives be provided for all medical students. The efforts outlined above have centered on trainees in formal public health programs, however we recognize that reduction in health disparities will require a more interdisciplinary approach, with a more active interface between public health programs and health professional schools (22). There is renewed optimism in achieving the goal of expanding a diverse public health workforce, based on the Liaison Committee on Medical Education (LCME) standards for public health science instruction and the ongoing initiatives in public health instruction throughout US medical schools (14).

The AAMC and LCME data (19) indicate that many U.S. medical schools now report a broader range of population-based medicine topics in their curriculum, such as biostatistics, epidemiology, health disparities, health care financing, health care quality improvement, public health systems, and global health issues. Prevention and health maintenance is also a required topic of instruction in most medical schools. In June 2010, the LCME, the body that sets accreditation standards for MD-granting medical schools, revised two of their educational standards (ED-11, ED-15) (23) to explicitly include public health sciences and preventive medicine.

## ED-11

The curriculum of a medical education program must include content from the biomedical sciences that supports students’ mastery of the contemporary scientific knowledge, concepts, and methods fundamental to acquiring and applying science to the health of individuals and populations and to the contemporary practice of medicine.

## ED-15

The curriculum of a medical education program must prepare students to enter any field of graduate medical education and include content and clinical experiences related to each phase of the human life cycle that will prepare students to recognize wellness, determinants of health, and opportunities for health promotion; recognize and interpret symptoms and signs of disease; develop differential diagnoses and treatment plans; and assist patients in addressing health-related issues involving all organ systems.

## Review of Medical School Best Practices

The University of New Mexico School of Medicine Public Health Certificate – The first year of medical school begins with a public health course: “Health Equity: Principles of Public Health.” The courses includes didactic lectures, case-based discussions, and introduces the social determinants of health through the documentary “Unnatural Causes: Is Inequality Making Us Sick?” (24). During the clinical years, the Objective Structured Clinical Examinations (OSCEs) are modified to evaluate students using policy and patient vignettes that weave public health issues into patient histories. Community-based service learning is integrated over the 4-year curriculum, in which students examine community health issues. They identify factors that exist in the community that affect the health of its residents and then undertake to develop solutions.

University of Wisconsin (UW) School of Medicine and Public Health – In 2005, the UW Medical School sought and received approval from the UW Board of Regents to change its name to the UW School of Medicine and Public Health. The goal of the name change and the transformation that is being launched will bridge the disciplines of biomedical and population health sciences and integrate public health into the School's core mission. By doing this, a revolutionary new model will be developed, which will unite clinical medicine and public health. The overarching vision is extremely important, and admittedly ambitious: “We will build a new and better infrastructure for the promotion of health and the prevention, diagnosis, and treatment of disease for the people of Wisconsin, which will then serve the nation as the leading model for improving the health of the public.”

Washington University School of Medicine – Students are introduced to health care and public health in St. Louis through a series of didactic lectures, group teaching, and guided community tours. Afterward, case-based discussions are provided to first and second-year medical students enrolled in the Public Health Selective and the Practice of Medicine course. The cases integrate fundamentals of the social determinants theory and incorporate goals highlighted in the Society of General Internal Medicine Health Disparities Task Force (25). The cases promote an understanding of the social determinants of health (7) and a sound grasp of the explanatory theory of health and related behavioral health theories (26). Case discussions are followed by a presentation on community health planning.

## Conclusion

Improving health equity will to a large extent depend on improving health literacy while enriching the nation with a talented cohort of graduates from medical school and schools of public health who are well educated in the social determinants of health, and are vested with the financial resources to conduct cutting edge research in the area. Public Health Education and Promotion Research can greatly aid this effort by promoting a competency-based public health curriculum in schools of medicine in collaboration with schools of public health. An educated, activated, and diverse workforce will emerge, well-suited to tackle the persistent health disparities in the US and globally. In the landmark turn of the twentieth century report on medical education in the US, Abraham Flexner advocated the integration of public health and medical education when he identified at least three public-health-oriented principles that are repeated throughout his report (27) and contributed to his arguments for medical education reform:

- The training, quality, and quantity of physicians should meet the health needs of the public.
- Physicians have societal obligations to prevent disease and promote health, and medical training should include the breadth of knowledge necessary to meet these obligations.
- Collaborations between the academic medicine and public health communities result in benefits to both parties.

After more than a century, Flexner's arguments resonate stronger than ever. Hopefully, through a comprehensive, interdisciplinary approach, we can bridge the irrational divide between medical education and public health and eliminate health disparities once and for all.
